# Role of *CYP9E2* and a long non-coding RNA gene in resistance to a spinosad insecticide in the Colorado potato beetle, *Leptinotarsa decemlineata*

**DOI:** 10.1371/journal.pone.0304037

**Published:** 2024-05-24

**Authors:** Emine Kaplanoglu, Ian M. Scott, Jessica Vickruck, Cam Donly

**Affiliations:** 1 London Research and Development Centre, Agriculture and Agri-Food Canada, London, ON, Canada; 2 Department of Biology, University of Western Ontario, London, ON, Canada; 3 Fredericton Research and Development Centre, Agriculture and Agri-Food Canada, Fredericton, NB, Canada; Institute of Zoology Chinese Academy of Sciences, CHINA

## Abstract

Spinosads are insecticides used to control insect pests, especially in organic farming where limited tools for pest management exist. However, resistance has developed to spinosads in economically important pests, including Colorado potato beetle (CPB), *Leptinotarsa decemlineata*. In this study, we used bioassays to determine spinosad sensitivity of two field populations of CPB, one from an organic farm exposed exclusively to spinosad and one from a conventional farm exposed to a variety of insecticides, and a reference insecticide naïve population. We found the field populations exhibited significant levels of resistance compared with the sensitive population. Then, we compared transcriptome profiles between the two field populations to identify genes associated primarily with spinosad resistance and found a cytochrome P450, *CYP9E2*, and a long non-coding RNA gene, *lncRNA-2*, were upregulated in the exclusively spinosad-exposed population. Knock-down of these two genes simultaneously in beetles of the spinosad-exposed population using RNA interference (RNAi) resulted in a significant increase in mortality when gene knock-down was followed by spinosad exposure, whereas single knock-downs of each gene produced smaller effects. In addition, knock-down of the *lncRNA-2* gene individually resulted in significant reduction in *CYP9E2* transcripts. Finally, *in silico* analysis using an RNA-RNA interaction tool revealed that *CYP9E2* mRNA contains multiple binding sites for the *lncRNA-2* transcript. Our results imply that *CYP9E2* and *lncRNA-2* jointly contribute to spinosad resistance in CPB, and *lncRNA-2* is involved in regulation of *CYP9E2* expression. These results provide evidence that metabolic resistance, driven by overexpression of CYP and lncRNA genes, contributes to spinosad resistance in CPB.

## Introduction

Spinosad is a naturally-derived insecticide composed of two tetracyclic macrolide molecules, spinosyn A and spinosyn D [1]. These insecticide molecules are produced by a soil bacterium, *Saccharopolyspora spinosa*, through fermentation, and their mode of action primarily involves allosteric interactions with nicotinic acetylcholine receptors (nAChRs), in addition to having an antagonistic effect on γ-aminobutyric acid (GABA) receptors [2, 3], leading to involuntary muscle contractions and subsequent paralysis and death of exposed insects. Being of natural origin [1], exhibiting low toxicity to mammals [4, 5] and having high selectivity towards specific insects [6], spinosad is considered to be safer than synthetic insecticides, and is approved for use in both organic and conventional farming in many countries against multiple insect pest orders, including Lepidoptera, Hemiptera, Diptera, and Coleoptera [7].

Spinosad insecticide is effective by both contact and ingestion, and has become a mainstay for controlling many pests. While this insecticide still continues to be effective against many insects, the emergence of resistance has been on the rise. For instance, moderate to high level spinosad resistance has been observed in multiple field populations of insect pests, including in tomato borer *Tuta absoluta* M. [8], onion thrips *Thrips tabaci* L. [9], olive fruit fly *Bactrocera oleae* R. [10], diamondback moth *Plutella xylostella* L. [11], melon fly *Zeugodacus cucurbitae* C. [12], western flower thrips *Frankliniella occidentalis* P. [13] and Colorado potato beetle (CPB) *Leptinotarsa decemlineata* S. [14]. This is of great concern for the future of pest management strategies for crop protection, especially for organic farmers that exclusively rely on naturally-derived insecticides.

Several studies have shown that spinosad resistance in insects can be caused by target-site insensitivity or metabolic resistance [15]. For example, mutations resulting in single amino acid substitutions or production of truncated nAChRα6 subunit protein have been shown to confer spinosad resistance in *F*. *occidentalis* [16], *P*. *xylostella* [17], and oriental fruit fly *Bactrocera dorsalis* H. [18]. In addition, metabolism or sequestration of spinosad molecules by esterase enzymes also has been implicated in some insects [19, 20]. The contribution of glutathione S-transferases (GSTs), another class of insecticide metabolizing enzymes, to spinosad resistance was minor as demonstrated by synergism studies [21, 22]. Primarily, previous studies indicate that metabolic resistance to spinosad is conferred by cytochrome P450 enzymes (CYPs) [23, 24], which comprise a superfamily of enzymes capable of performing a broad range of functions, including insecticide metabolism [25]. For instance, multiple CYP genes were found to be overexpressed in spinosad-resistant housefly *Musca domestica* L., *T*. *tabaci* and *P*. *xylostella* [26–28]. Similarly, exposure to spinosad results in upregulation of CYP genes in the CPB [29, 30].

In most cases, CYP-mediated insecticide resistance is caused by overexpression of one or more CYP enzymes, either constitutively or upon insecticide exposure [31], allowing resistant insects to break-down the insecticides more effectively compared with sensitive insects. Oftentimes, over-expression of CYPs is caused by changes in *cis*-or-*trans*-acting regulatory loci and gene duplications. For example, mutations in promoter regions (*cis*-acting), gene amplifications and changes in *trans*-acting regulator expression, such as Nrf2/Keap1 pathway, all lead to overexpression of CYPs in multiple insects, subsequently resulting in insecticide resistance [32–34].

In recent years, studies have also elucidated the role of non-coding RNA (ncRNA) species in regulation of CYP expression and their contribution to insecticide resistance [35–38]. These non-translated RNA molecules include micro RNAs (miRNAs, < 200 nt long) and long non-coding RNAs (lncRNAs, > 200 nt long). While miRNAs primarily regulate gene expression by triggering post-transcriptional degradation of target mRNA or translational arrest [39], lncRNAs are known to regulate gene expression at both the post-transcriptional and transcriptional levels by acting as miRNA precursors or miRNA sponges and by binding to transcription factors and removing them from chromatin [40]. In addition, lncRNA can directly bind and stabilize target mRNA, resulting in more efficient translation [41]. In many insecticide resistant insects, miRNA and lncRNA are differentially expressed, which, in turn, results in up/down regulation of CYPs and other resistance-related genes. For instance, multiple lncRNAs, that were strongly associated with CYPs, GSTs and UDP-glycosyltransferases (UGTs), were up/down regulated in malathion resistant *B*. *dorsalis* and cyflumetofen resistant carmine spider mite *Tetranychus cinnabarinus* B. and associated with resistance to nicotine in tobacco aphid *Myzus persicae nicotianae* B. [37, 42, 43]. Similarly, exposure to spinosad results in upregulation of multiple miRNAs in CPB [30].

In this study, our objective was to identify molecular mechanisms of spinosad resistance in CPB, which is a major pest of potato plants causing significant crop losses [44]. Spinosad has been used to control this pest for more than two decades; however, current reports of control failure due to resistance development in multiple field populations have raised great concern [14, 45, 46]. Although there are currently a few studies showing possible involvement of CYPs and miRNAs in spinosad resistance in CPB [29, 30], there is still a large knowledge gap in understanding the molecular mechanism of resistance and the specific genes involved. To address some of this gap, we first showed the presence of spinosad resistance in two field populations of CPB having different histories of insecticide exposure, compared with an insecticide naïve laboratory population. This was followed by comparison of the transcriptome profiles of the two field populations to identify constitutively differentially expressed insecticide detoxifying and lncRNA genes in the field population exposed to spinosad only. Finally, we used RNA interference (RNAi) combined with insecticide exposure to determine if differentially expressed genes have a role in the spinosad resistance observed in these insects.

## Methods

### CPB populations and rearing conditions

Two different field CPB populations and an insecticide naïve laboratory population were used in this study. The insecticide sensitive laboratory population (SLP) was originally collected from a potato field at the London Research and Development Centre, London, Ontario, Canada, and has been in continuous culture over 220 generations without insecticide exposure. This population has never been exposed to spinosad insecticides, and it has been used as a sensitive population in previous studies [45, 47]. The two field populations were collected from potato farms in the province of Québec, Canada. One of these field populations, here referred to as “organic farm population”, OFP, was collected from an organic potato farm in Farnham, Québec, where only spinosad was used for pest control. The second field population, here referred to as “conventional farm population”, CFP, was collected from a potato farm in Saint-Leonard d’Aston, Québec, where spinosad and other insecticides, including neonicotinoids, were used for pest control. The beetles were collected by the potato growers and shipped to the researchers’ laboratories; therefore no field access permits were required for this study. After reception, the beetles were reared on potato plants (*Solanum tuberosum* var. Kennebec) in screen cages at 25°C, 50% relative humidity (RH), and 16: 8 h light: dark photoperiod following previously described methods [48]. Beetles from each population were housed in separate screen cages in the same environmental growth room without any insecticide exposure.

### Bioassays to determine LD_50_ of spinosad in CPB populations

Spinosad (Entrust^®^) was obtained from Corteva Agriscience Canada (Calgary, AB, Canada). Entrust contains 22.5% active ingredient spinosad (Qalcova^®^) in its formulation. Serial dilutions were prepared (S1 Table for concentrations) using sterile deionized water, and 1 μL of insecticide solution was placed on a 5 mm disk punched out of a potato leaf and allowed to dry. Mixed-sex adults, that were less than 7-days old, were starved for 3–4 h and each insect was then provided with an insecticide treated leaf disk. Insects were allowed to feed on the leaf disk overnight, and only the ones that consumed the entire disk were included in the bioassays to ensure all insects received the given dose. After insecticide dosing, insects were provided with untreated potato leaves and allowed to feed *ad libitum* for seven days, and death and intoxication were recorded daily. After seven days, the intoxicated insects (unable to right themselves or unable to walk a distance equivalent to their body length) were regarded as moribund and counted as dead when analysing the data. At least 29–30 insects per dose were used, and probit analysis was used to estimate the spinosad dose needed to cause 50% mortality (LD_50_) in the three CPB populations. Resistance ratios were calculated using the LD_50_ of OFP or LD_50_ of CFP divided by the LD_50_ of SLP. LD_30_ of the most resistant population was also calculated to use in the subsequent RNAi and spinosad exposure bioassays.

### RNA extraction and RNA sequencing (RNA-seq)

RNA-seq was used to identify differentially expressed genes between the two field populations to selectively identify genes associated with spinosad resistance specifically. We did not use SLP in RNA-seq to avoid comparing a laboratory population to field populations, which could lead to detection of very high numbers of differentially expressed genes simply due to differences in life histories of the insects [26, 28]. For RNA extractions, ≤ 2 day-old mixed-sex adults from OFP and CFP populations were used. Insects were dissected in Calpode’s insect saline (pH = 7.2, 10.7 mM NaCl, 25.8 mM KCl, 90 mM glucose, 29 mM CaCl_2_, 20 mM MgCl_2_ and 5 mM HEPES), and midgut, Malpighian tubule, and fat body tissues were isolated. The three tissues were pooled, immediately suspended in RNAlater buffer (Thermo-Fisher, Waltham, MA, USA) and stored at -80°C until RNA extraction. Tissues from four beetles were combined to form a biological replicate, and three biological replicates were done for each population. To extract total RNA, mirVana miRNA Isolation Kit (Thermo-Fisher) was used. Tissues were removed from RNAlater buffer and homogenized in Lysis/Binding Buffer using a Polytron Handheld Homogenizer. Total RNA was extracted using the protocol described in the kit’s manual. Then, total RNA samples were diluted to 150 ng/μL using nuclease-free water, and 1 μL was used to assess integrity of RNA using a 2100 Bioanalyzer (Agilent, Mississauga, ON, Canada). Fifteen μL of the diluted samples were shipped on dry ice to Génome Québec (Montréal, QC, Canada) for sequencing. mRNA library construction, using NEB mRNA stranded library preparation, Illumina library QC, and sequencing of libraries on the Illumina NovaSeq 6000 platform were performed at Génome Québec, using the 100 bp paired-end protocol (Illumina, San Diego, CA, USA).

### RNA-seq to identify differentially expressed sequences between OFP and CFP and molecular pathway enrichment analyses

Sequence data was imported to CLC Genomics Workbench version 20.0.1 (Qiagen Bioinformatics, Germantown, MD, USA), and quality control (QC), which included quality trim, ambiguous base trim, adapter trim and homopolymer trim, was performed using the software’s default parameters. Reads having < 20 nt after trimming were discarded, followed by mapping of the trimmed reads to a reference Colorado potato beetle transcriptome, lepdec_OGSv1.1(downloaded from https://data.nal.usda.gov/dataset/leptinotarsa-decemlineata-official-gene-set-v11) [49], which contains a total of 24,935 transcripts. For mapping, default parameters (80% similarity over 80% of length, with two mismatches allowed) were used. To account for differences between sequencing depth among samples, library sizes were normalized using trimmed mean of M values (TMM). Gene expression was quantified using transcripts per million (TPM), and the transcripts were considered differentially expressed if the absolute value of log_2_Fold change was ≥ 1 and adjusted *P*-value (*P*-adj) was ≤ 0.05 after the Benjamini-Hochberg false discovery rate (FDR) correction [50]. Finally, differentially expressed sequences between OFP and CFP populations were screened manually to identify sequences encoding insecticide detoxifying enzymes including cytochrome P450s (CYPs), GSTs, UGTs, esterases or lncRNAs, with constitutively increased transcript levels.

Finally, once differentially expressed sequences were identified, ShinyGO 0.9 (http://bioinformatics.sdstate.edu/go/) [51] was used to determine molecular pathways enriched in the two field populations.

### cDNA synthesis and RT-qPCR to confirm differentially expressed genes

To confirm RNA-seq results, independent biological samples were used to perform RT-qPCR for four selected genes, three from the RNA-seq results and one additional lncRNA gene, a gene that was upregulated in a CPB population from Ontario, Canada showing resistance to spinosad, as well as two diamide insecticides [45]. Expression of the four genes was also analyzed in the SLP to determine whether changes in gene expression for the four target genes correlated with resistance levels. For this purpose, total RNA was extracted from adult beetles as described previously, and RNA samples were treated with Ambion Turbo RNase-Free DNase kit (Thermo-Fisher) using manufacturer’s instructions to remove contaminating gDNA. Then, cDNA was synthesized from 1 μg of total RNA using Invitrogen Superscript III First-Strand Supermix Kit (Thermo-Fisher) following the manufacturer’s protocol. RT-qPCR reactions were performed using a SensiFAST SYBR No-ROX Mix Kit (Meridian Bioscience, Memphis, TN, USA), forward and reverse primers at 400 nM each and 2.5 μL of a 1: 3 dilution of cDNA template in 10 μL final reactions using a CFX96 Real-Time Detection System (Bio-Rad, Mississauga, ON, Canada). Three reference genes, ribosomal protein (*L8E*), ADP-ribosylation factor 1 (*ARF1*), and translation elongation factor 1α (*EF1α*), were used to normalise transcript abundance of target genes. All RT-qPCR primers were validated to ensure compliance with minimum information for publication of quantitative real-time PCR experiment guidelines [52]. All primers are listed in S2 Table.

### Double-stranded RNA production for gene knock-down using RNAi

Double-stranded RNA (dsRNA) production was accomplished using previously published methods [53–55]. Briefly, dsRNA fragments targeting *lncRNA-2* and *CYP9E2* were selected using E-RNAi web tool [56], followed by cloning of selected fragments into pL4440 vector using restriction enzyme cloning with a T4 DNA ligase kit (Thermo-Fisher). The ligation reactions were then transformed into *Escherichia coli* HT115 using a standard heat shock transformation protocol. The cells were grown on Luria Bertani (LB) agar plates containing ampicillin (100 μg/mL) and tetracycline (12.5 μg/mL). PCR was used to screen positive colonies, from which plasmids were extracted for sequencing to confirm the presence of target sequences in the plasmids. In addition to the two target genes, a 449 nt long *Green Fluorescent Protein* (*GFP*) dsRNA was also produced in *E*. *coli* HT115 cells and was used as a dsRNA control.

To induce *E*. *coli* HT115 for dsRNA production, cells were inoculated in LB medium containing ampicillin (100 μg/mL) and tetracycline (12.5μg/mL). The cultures were grown until the optical density at 600 nm (OD_600_) was 0.4–0.6. At this point, isopropyl β-D-1-thiogalactopyranoside (IPTG) was added to the culture at a final concentration of 1 mM to induce production of dsRNA in the *E*. *coli* HT115 cells. The cultures were grown for an additional 4 h after the addition of IPTG. On average, after 4 h of induction with IPTG, the OD_600_ of cells was 1.123 (5.62 × 10^8^ cells/mL). The cells were centrifuged 8,000 g for 10 min at 4°C, and the resulting pellets were washed once with 1 × PBS (pH = 7.4, 137 mM NaCl, 2.7 mM KCl, 10 mM Na2HPO4, 2 mM KH2PO4). Finally, pellets were re-suspended in 1 × PBS buffer to concentrate the initial culture 10 × yielding a concentration of 5.62 × 10^9^ cells/mL, which was then used to dip potato leaves for gene knock-downs. Production of dsRNA was confirmed by extracting total nucleic acids from 1 mL of un-concentrated cultures using a MasterPure Complete DNA and RNA Purification Kit (Illumina), followed by removal of ssRNA and DNA using T7 RiboMax express RNAi system Kit components and the kit protocol (Promega, Madison, WI, USA). Finally, a Qiagen RNeasy kit (Qiagen) was used to further purify the dsRNA. Purified dsRNA was quantified using a Nanodrop 1000 spectrophotometer (Thermo-Fisher), which suggested that on average, there were about 2.85 μg of dsRNA in one mL of un-concentrated *E*. *coli*. Also, a 700 ng aliquot of dsRNA was visualized on an agarose gel to confirm dsRNA integrity (S1 Fig).

### RNA interference followed by insecticide exposure

RNAi of target genes was accomplished by feeding OFP beetles with either 1 × PBS buffer only, or *E*. *coli* HT115 cells producing dsRNA for target genes or *GFP*. Briefly, potato leaves were dipped in bacterial cultures or buffer and were allowed to dry on a metal mesh under airflow for 1 h. Then, one treated leaf was placed into a petri dish lined with moistened Whatman filter paper. OFP beetles, that were ≤ 24 h old, were starved for 3–4 h, and one beetle was put in each Petri dish. The beetles fed on the treated leaves *ad libitum* for four days and were provided with freshly treated leaves daily. For double-knock-down, *E*. *coli* HT115 cultures producing dsRNA for each of the genes were mixed in 1: 1 ratio and fed to the insects. After four days of dsRNA feeding, four insects per biological replicate were randomly selected and were dissected for RNA extractions and RT-qPCR to confirm gene knock-down. Remaining insects were exposed to ~ LD_30_ of spinosad using the leaf disk method as described in previous sections. A lower dose equal to ~LD_30_ was used instead of ~LD_50_, since beetles have reduced tolerance for spinosad after knock-down of genes promoting resistance. Insects that consumed the whole disk containing insecticide were then provided with untreated potato leaves and monitored daily for seven days to assess mortality.

### Role of *lncRNA-2* in *CYP9E2* regulation

Beetles were fed 20 × concentrated *E*. *coli* producing *lncRNA-2* dsRNA for four days, and *CYP9E2* transcript levels were analyzed using RT-qPCR as described previously, to see if knock-down of *lncRNA-2* had any impact on *CYP9E2* mRNA levels. In addition, *in silico* analysis of *lncRNA-2* and *CYP9E2* mRNA interaction was performed using the default parameters in the IntaRNAv2 algorithm (Freiburg RNA tools: http://rna.informatik.uni-freiburg.de/IntaRNA) [57]. The software provides minimal energy profiles for RNA–RNA interaction sites in a given pair, and the lower the energy required, the stronger the interaction is predicted to be.

### Statistical analysis

Probit analysis was used to calculate LD_50_ of spinosad for SLP, OFP and CFP populations [58]. The populations that had no overlap between the 95% fiducial limits of LD_50_ were considered to have different resistance levels to spinosad as described previously [59]. RT-qPCR results were analyzed using the 2^-ΔΔCt^ method utilizing Bio-Rad CFX Maestro software version 2.2, while the statistical analyses of the data were done using two sample *T*-tests or one-way ANOVA followed by Tukey’s HSD *post hoc* analysis where applicable, using R version 4.3.1 [60]. Finally, Kaplan-Meier survival curves and Log-rank tests were performed using survival package in R to identify differences in survival in insects exposed to different treatments. A *P*-value of ≤ 0.05 was used as significance level in all analyses.

## Results

### Spinosad resistance in CPB populations

Results for LD_50_ estimations of spinosad in three CPB populations are summarized in Table 1. Results showed that there were no overlaps between the 95% fiducial limits of LD_50_ values which indicates that the resistance levels are different among the populations. Both field populations had higher LD_50_ values for spinosad compared with the SLP, with resistance ratios of 12.10 × and 4.05 × for OFP and CFP, respectively. The resistance ratio between the two field populations was also calculated, and results showed that OFP had an almost three-fold increase in spinosad resistance compared with CFP. As OFP was the most resistant to spinosad, this population was selected for future studies using RNAi, followed by insecticide exposure. For this purpose, LD_30_ of spinosad was used which was determined to be 3.0 μg/beetle.

**Table 1:** Spinosad bioassays to determine LD_50_ in three CPB populations.

Population	n^a^	df^b^	LD_50_ (μg/beetle)	95% fiducial limit	Slope (±SE)	χ2	Resistance Ratio (RR)^c^
**Sensitive Laboratory (SLP)**	245	5	0.41	(0.28, 0.59)	1.97 (0.08)	1.00	1.00
**Conventional Farm (CFP)**	289	6	1.66	(1.25, 2.20)	2.69 (0.06)	0.93	4.05
**Organic Farm (OFP)**	476	11	4.96	(3.71, 6.64)	1.90 (0.06)	0.45	12.10

^a^Total number of insects tested

^b^degrees of freedom

^c^RR was calculated by dividing LD_50_ of CFP or OFP with LD_50_ of SLP

### Differentially expressed genes between CFP and OFP populations using RNA-seq

In total, mRNA sequencing of 6 libraries yielded more than nine hundred million reads. Overall, high quality reads mapping to a previously published transcriptome [49] in pairs and in broken pairs per sample ranged from 58.72% to 59.38% and 7.83% to 8.35%, respectively (S3 Table). Mapped reads were then used to identify differentially expressed sequences between the two populations using Genomics Workbench software (Qiagen Bioinformatics). Between CFP and OFP, 200 differentially expressed transcripts were identified (S2 Fig), of which, 101 were upregulated, and 99 were downregulated in OFP. Then, differentially expressed transcripts were manually screened to identify genes encoding insecticide detoxifying enzymes and lncRNA genes. Four CYP encoding transcripts, LDEC021333-RA, LDEC021334-RA, LDEC022309-RA and LDEC013538-RA were up-regulated, and of those, the first three corresponded to *CYP9E2*-like gene (*CYP9E2*, LOC111507098, XM_023162346.1) while the fourth transcript corresponded to a probable *CYP6A23* gene (*CYP6A23*, LOC111510688, XM_023166624.1). Of the 99 down-regulated transcripts, three were CYP genes (*CYP6K1-like*, *CYP6A13* and *CYP412A1*). With respect to lncRNA genes, only one lncRNA transcript, LDEC021826-RA (LOC111516474, XR_002723681.1) was upregulated, and it is referred to as *lncRNA-1* in this study. Also, three lncRNA transcripts (LDEC019091-RA, LDEC004225-RA and LDEC002474-RA) were downregulated. In terms of other insecticide metabolizing enzymes, such as GSTs, UGTs or esterases, no transcripts were constitutively differentially expressed. A full list of transcripts showing differential expression based on an FDR value of 0.05 significance level between CFP and OFP is shown in S4 Table.

In addition, ShinyGO analysis revealed that molecular pathways involved in catalytic activity, glutamate receptor activity, calcium channel activity, hydrolase activity, multiple ion transport, nuclease activity, peptidase activity and passive transmembrane transporter activity were among the enriched pathways in OFP (S3A Fig). In contrast, molecular pathways involved in lipid transport, oxidoreductase activity and flavin adenine dinucleotide binding were enriched in CFP (S3B Fig).

### RT-qPCR confirmation of RNA-seq results

*CYP9E2*, *CYP6A23* and *lncRNA-1* (all up-regulated in OFP compared with CFP based on RNA-seq analysis) genes were selected for confirming RNA-seq results. In addition, transcript levels of another lncRNA gene, referred to as *lncRNA-2* in this study (*lncRNA-2*, LOC111518075, XR_002723912.1), was also analyzed using RT-qPCR, as this particular gene was up-regulated in a CPB population from Ontario, Canada showing resistance to spinosad and two diamide insecticides [45], as RNA-seq can sometimes miss differentially expressed genes [61].

**Fig 1:**
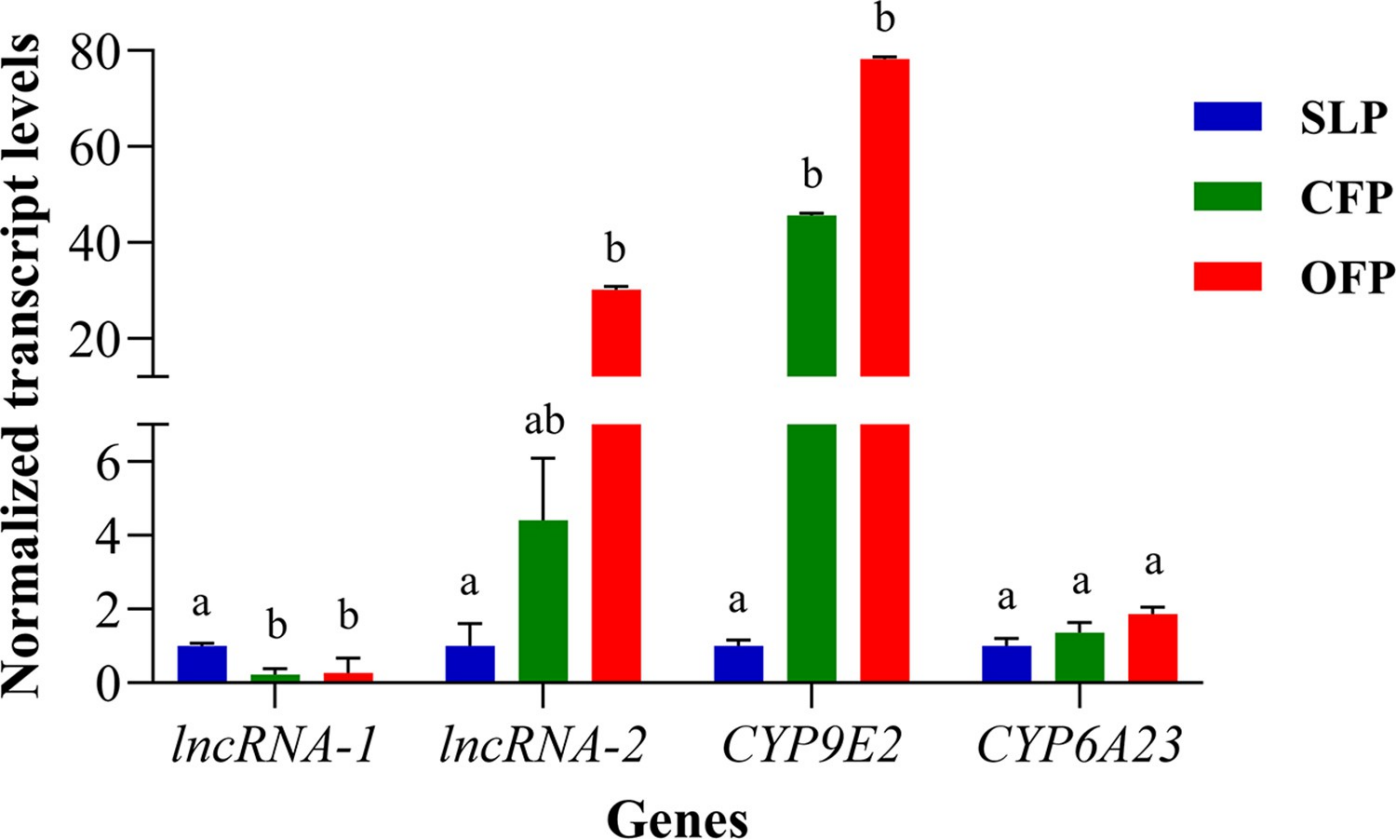
RT-qPCR to confirm RNA-seq results in independent biological samples. Normalized transcript level in SLP population was set to “1”, and fold changes in OFP and CFP were calculated compared with levels in SLP using 2^-ΔΔ Cq^ method. Data are presented as mean relative quantity ± SEM, n = 3. Letters placed above bars denote significant differences in mRNA levels for each gene. Means with the same letter are not significantly different (*P* > 0.05) according to Tukey’s HSD test (one-way ANOVA).

We found that *lncRNA-1*, *lncRNA-2*, *CYP9E2* and *CYP6A23* mRNA levels are elevated in OFP compared with CFP, although RNA-seq and RT-qPCR detected different fold changes in mRNA levels (S5 Table). When the results from RT-qPCR were analyzed using one-way ANOVA tests to compare the mRNA levels of target genes among the three populations, *CYP9E2* was shown to be significantly upregulated in both field populations compared to SLP (*F*_2,6_ = 70.86, *P* < 0.001), while the difference between OFP and CFP was not significant (*P* > 0.05). mRNA levels of *lncRNA-2* were also significantly upregulated in OFP compared with SLP (*F*_2,6_ = 4.903, *P* = 0.05), however, the differences between OFP and CFP and between SLP and CFP were not significant (*P* > 0.05). Interestingly, mRNA levels of *lncRNA-1* were downregulated in both OFP and CFP compared with SLP (*F*_2,6_ = 20.34., *P* = 0.0020), while the difference between OFP and CFP was not significant (*P* > 0.05). Furthermore, no change in expression of *CYP6A23* was observed among the three populations (*P* > 0.05) (Fig 1).

### Phenotypic effects of individual gene knock-downs on spinosad resistance in OFP

**Fig 2:**
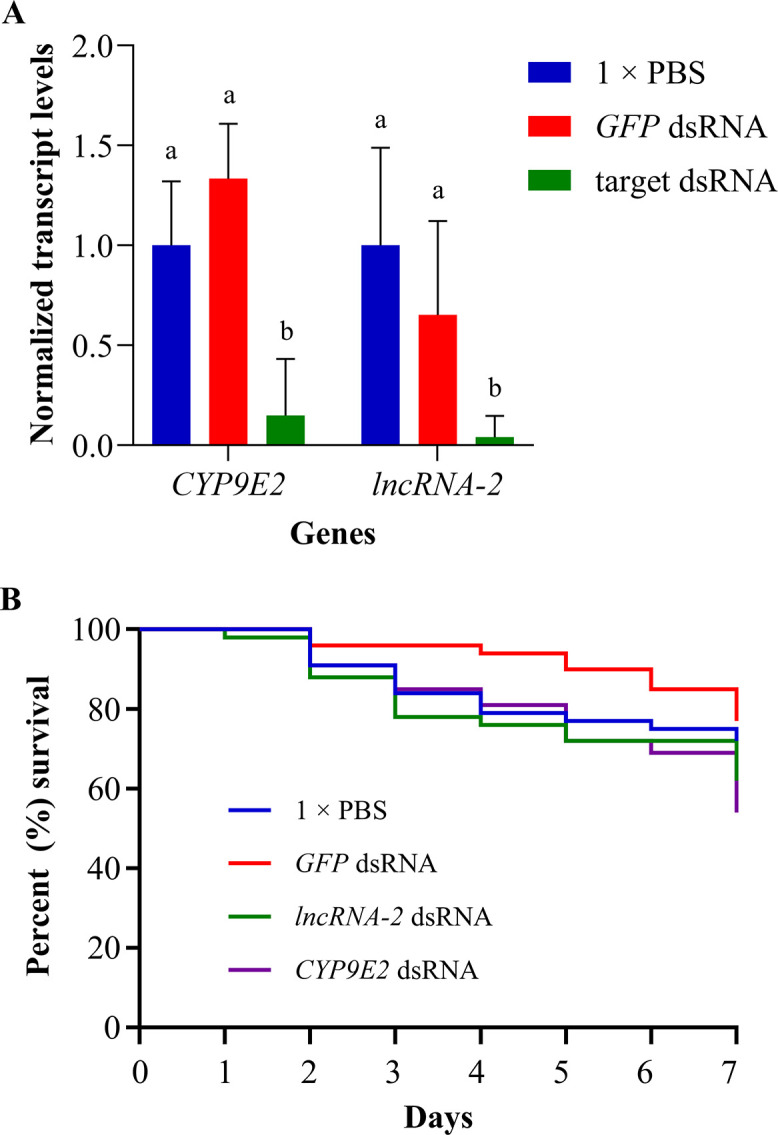
RT-qPCR results for individual knock-down of *CYP9E2* and *lncRNA-2* genes using RNAi and survival of OFP adults following insecticide exposure. (A) Normalized transcript levels in PBS-fed OFP was set to the value of “1.0”, and transcript levels in GFP dsRNA and target dsRNA-fed OFP were calculated relative to the PBS-fed OFP. Data are expressed as mean relative quantity ± SEM, n = 4. Letters placed above bars denote significant differences in transcript levels for each gene, and means with the same letter are not significantly different (*P >* 0.05) according to Tukey’s HSD (one-way ANOVA). (B) Kaplan-Meier survival curves illustrating the percent survival of OFP beetles after an LD_30_ challenge of spinosad insecticide. Beetles were provided with potato leaves treated with 1 × PBS (n = 81), *GFP* dsRNA (n = 52), *CYP9E2* dsRNA (n = 54) or *lncRNA-2* dsRNA (n = 50) for four days, followed by exposure to LD_30_ of spinosad (3 μg/beetle) on leaf disks.

OFP was selected for RNAi followed by spinosad exposure, as this population had the highest levels of mRNA transcript for the selected genes (*CYP9E2* and *lncRNA*-2), implying that upregulation of these genes could be contributing to resistance. The results showed that transcript levels were reduced 85.1%, and 95.9% for *CYP9E2* and *lncRNA-2*, respectively, in target dsRNA-fed beetles compared with 1 × PBS control. One-way ANOVA tests confirmed that feeding CPB dsRNA for *CYP9E2* and *lncRNA-2* genes resulted in a significant reduction in the transcript levels for both genes (F_2,9_ = 34.94, *P* < 0.001 for *CYP9E2* and F_2,9_ = 7.38, *P* = 0.013 for *lncRNA-2*). Tukey’s honest significant difference (HSD) tests showed that transcript levels of *CYP9E2* and *lncRNA-2* in *GFP* dsRNA-fed CPB were not significantly different from transcript levels in PBS-fed CPB (*P* > 0.05), confirming gene knock-down was specific for these two targets (Fig 2A).

After gene knock-down, OFP beetles were exposed to LD_30_ of spinosad to determine whether spinosad toxicity would increase upon gene knock-down. Mortality of OFP beetles after seven days was 28.4%, 23.1%, 46.3% and 38.0% in 1 × PBS, *GFP* dsRNA, *CYP9E2* dsRNA and *lncRNA-2* dsRNA fed beetles, respectively (Fig 2B). Analysis of results with Kaplan-Meier survival curves and Log-rank tests showed that, although there was an increase in mortality in beetles fed *CYP9E2* dsRNA and *lncRNA-2* dsRNA, the results were not statistically significant (*P* > 0.05).

### Simultaneous knock-down of *CYP9E2* and *lncRNA-2* and its effect on spinosad resistance

**Fig 3:**
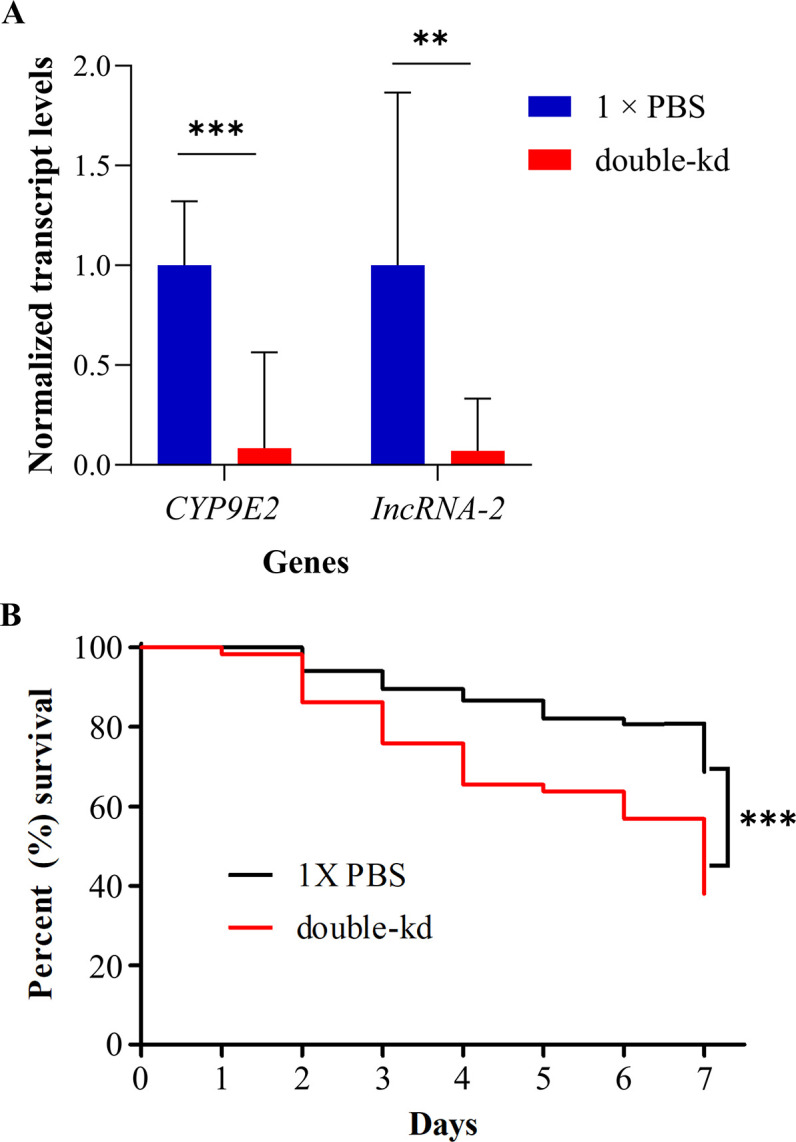
Gene knock-down and survival bioassays after simultaneous knock-down of *CYP9E2* and *lncRNA-2*. (A) RT-qPCR results for simultaneous knock-down of *CYP9E2* and *lncRNA-2* genes. Beetles were provided with a 1: 1 ratio of bacteria producing dsRNA targeting the two genes for four days. Normalized transcript levels were set to the value of “1” in 1 × PBS control and the differences in transcript levels in dsRNA-fed insects were calculated relative to control. Data is presented as mean relative quantity ± SEM. Asterisks represent significant changes in *t*-tests (***P ≤ 0.001 and ** *P* ≤ 0.01), n = 4. (B) Kaplan-Meier survival curves illustrating the percent survival of the double-kd OFP beetles after an LD_30_ challenge of spinosad insecticide. Beetles were provided with potato leaves treated with 1 × PBS (n = 67) or 1: 1 ratio of bacteria producing dsRNA for *CYP9E2* and *lncRNA-2* (n = 58) for 4 days, followed by exposure to LD_30_ of spinosad (3 μg/beetle) on leaf disks. Double-kd = double-knock-down.

Simultaneous knock-down of *CYP9E2* and *lncRNA-2* resulted in significant down-regulation of both genes (Fig 3A), as *CYP9E2* was knocked-down by 91.7% (*t* = -6.29, *P* < 0.001), while *lncRNA-2* was knocked-down by 92.84% (*t* = -4.34, *P* = 0.0049). In addition, double knock-down of both genes resulted in a significant increase in mortality in OFP beetles after exposure to LD_30_ of spinosad (Fig 3B). In dsRNA-fed beetles, mortality increased to 62.1% as compared to 31.4% in 1 × PBS control. Kaplan-Meier survival curves followed by Log-rank tests showed that the results were significant (χ^2^ = 12.4, df = 1, *P* < 0.001).

### Effect of *lncRNA-2* gene knock-down on *CYP9E2* transcript level

**Fig 4:**
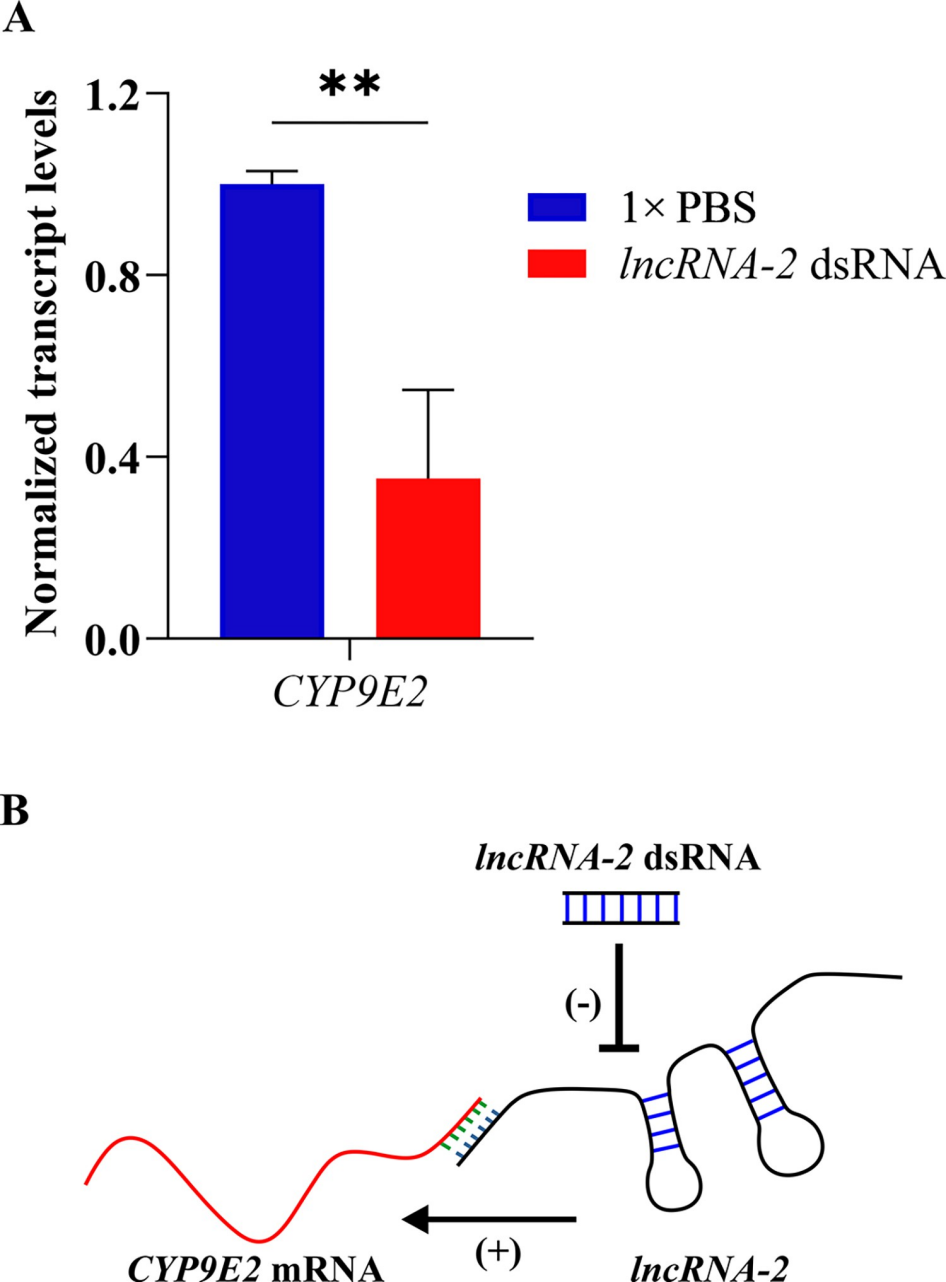
RT-qPCR results measuring transcript levels of *CYP9E2* in *lncRNA-2* dsRNA-fed OFP beetles, with a schematic model for *CYP9E2* gene regulation by *lncRNA-2*. (A) Normalized transcript levels were set to the value of “1” in 1 × PBS control and the differences in transcript levels in dsRNA fed insects were calculated relative to control. Data is presented as mean relative quantity ± SEM. ** *P* ≤ 0.01), n = 4. (B) Proposed model for *lncRNA-2* regulation of *CYP9E2* expression.

To investigate whether *CYP9E2* transcript levels are affected by those of *lncRNA-2*, *CYP9E2* transcripts were measured in OFP beetles fed with a high level of *lncRNA-2* dsRNA (20 × concentrated *E*. *coli*). Compared with expression levels in control 1 × PBS fed insects, the *CYP9E2* gene transcript was reduced significantly, by 70.6%, in *lncRNA-2* dsRNA-fed insects (*T* = 5.18, *P* = 0.002) (Fig 4A). In addition, to predict whether the *lncRNA-2* and *CYP9E2* mRNAs could interact directly, IntaRNAv2 software was used, which identified four possible interaction sites in the pair, with interaction free energy scores less than 0.0 (S6 Table). The strongest interaction was with nucleotides 1373–1426 of *CYP9E2* mRNA with energy score of -13.35, which implies strong interaction. These results suggest that *lncRNA-2* may regulate the *CYP9E2* gene based on a simple model as depicted in Fig 4B.

## Discussion

We found that two field populations of CPB were resistant to spinosad insecticide compared with a sensitive laboratory population. However, one population (organic farm–OFP), collected from a farm using exclusively certified organic pest control products, had higher levels of spinosad resistance (RR ~ 12 ×) compared with the population collected from a farm that also employed conventional synthetic insecticides (conventional farm–CFP), which had RR ~ 4.0 ×. Previously, it has been found that spinosad resistance is more common in insects collected from organic farms [14], and our results appear to be in line with such observations. This outcome, however, is not surprising as there is a limited number of naturally-derived products available to manage insect pests on organic farms, and overuse of these products can ultimately lead to rapid resistance development. In addition, the resistance ratio was almost 3-fold between the two field populations based on the respective LD_50_ values for spinosad insecticide, which is similar to resistance ratios previously reported in field collected, spinosad resistant, insect populations [10, 14, 62]. Taken together, these results highlight the importance of developing alternative tools that could be utilized by organic farmers.

One of the main mechanisms for the development of insecticide resistance is modulation in the expression of specific genes that confer resistance. Over time, repeated exposure to the same insecticide selects the individuals in a population carrying such changes, ultimately leading to control failures [25]. Such changes in gene expression were evident in our RNA-seq results, which showed 200 transcripts as constitutively differentially expressed in the OFP population compared with the CFP population. As the transcriptome analysis was done using only the field populations, the approach was focused on differences resulting from exposure to different insecticides, specifically spinosad, since the OFP population was exposed to this product exclusively. Members of the CYP gene family were among the 200 differentially expressed transcripts, with two examples showing increased (*CYP9E2* and *CYP6A23*) and three examples showing decreased (*CYP6K1-like*, *CYP6A13* and *CYP412A1*) transcript levels. This was expected, as multiple other studies have shown up/down regulation of CYPs in insecticide resistant insects [54, 63, 64].

While previous studies showed that upregulation of GSTs, UGTs and esterases also contributes to spinosad resistance in other insects [26, 28], we did not observe increased transcription levels for these gene families in our analysis; hence the role of these genes in spinosad resistance in CPB remains unknown. However, in OFP, several molecular pathways, including catalase activity, glutamate receptor activity, hydrolase activity, calcium ion channel activity, cysteine type peptidase activity and nuclease activity, were enriched (S3 Fig), similar to the results in other insects [65, 66]. Interestingly, in *M*. *domestica*, a weak interaction between spinosyn A and the voltage-gated calcium channel was also implied [67], and two transcripts coding for subunits of these channels were upregulated in spinosad resistant flies [26]. In our analysis, we observed a voltage-dependent calcium channel type A subunit alpha-1 being significantly upregulated (S4 Table), suggestive of the importance of ion transport in contributing to spinosad resistance alongside the insecticide metabolizing enzymes and other enriched molecular pathways. Furthermore, molecular pathways involved in lipid transport, flavin adenine dinucleotide binding and oxidoreductase activities were all downregulated in OFP (S3 Fig). Interestingly, oxidoreductase activity was also downregulated in *P*. *xylostella* resistant to spinosad [28], which implies reduced energy metabolism in resistant insects, which may help insects allocate some resources for responding to spinosad pressure.

Of the genes found to be upregulated based on RNA-seq, we selected the two CYP genes, *CYP9E2* and *CYP6A23*, and analyzed their expression in the field populations as well as in the sensitive laboratory population. Of the two upregulated CYPs, we were able to confirm upregulation of *CYP9E2* in both field populations compared with SLP; and in OFP, which has the highest resistance to spinosad, the *CYP9E2* gene had the greatest transcript level increase. Interestingly, *CYP9E2* was also previously shown to be constitutively overexpressed in a New York population of CPB with overall reduced baseline sensitivity to multiple insecticides, including spinosad [68], and knock-down of this gene resulted in increased susceptibility to the neonicotinoid insecticide clothianidin [69]. Also, *CYP9E2* transcript levels were elevated in another CPB population upon spinosad exposure [29, 30]. These findings all imply a role for this gene in spinosad resistance. In our study, knock-down of *CYP9E2* followed by spinosad exposure resulted in 46.3% mortality, which was considerably, albeit not significantly, higher than 1 × PBS control treated insects (only 28.4% mortality). This, indeed, provides further evidence that *CYP9E2* at least partially contributes to spinosad resistance in CPB.

Another notable finding in our study was the identification of multiple differentially expressed lncRNA genes in the spinosad-resistant population, OFP. Unlike CYP genes, whose involvement in insecticide resistance is well-established, lncRNA genes have just recently become a focal point in insecticide resistance studies, and their role in insecticide resistance is less well understood. In our study, we identified *lncRNA-2* was constitutively overexpressed in OFP. In fact, RT-qPCR results showed that *lncRNA-2* levels were the highest in OFP, compared with two other populations, which implies its elevated expression may play a role in increased resistance to spinosad. To investigate the role of *lncRNA-2* in resistance further, we successfully knocked-down its expression using RNAi and then exposed the beetles to spinosad. This increased CPB mortality to 38.0%, an approximate 10% increase from 1 × PBS control, which may infer a partial role of this gene in spinosad resistance. With respect to the other lncRNA gene, *lncRNA-1*, we found that the *lncRNA-1* transcript levels were in fact significantly downregulated in both spinosad-resistant populations when compared with SLP, despite the fact that its transcript levels were elevated in OFP compared with CFP. Thus, it is unclear whether downregulation of this gene in spinosad-exposed field populations plays a role in resistance, or whether such differences are simply caused by the differences in life histories of the populations.

Having identified trends toward increased spinosad toxicity resulting from individual knock-downs of *CYP9E2* and *lncRNA-2*, we decided to target these two genes simultaneously. This resulted in a synergistic effect and caused a significant increase (62.1%) in CPB mortality upon spinosad exposure (an increase of 30.7% from 1 × PBS control), which demonstrates a polygenic nature for spinosad resistance in CPB. Our findings are in line with a previous study which suggested that spinosad resistance was controlled by multiple loci in *M*. *domestica* [70]. lncRNAs are mainly thought to contribute to insecticide resistance by regulating other resistance-related genes. For instance, a lncRNA has been shown to positively regulate cadherin expression in pink bollworm *Pectinophora gossypiella* S., subsequently leading to *Bacillus thuringiensis* toxin Cry1Ac resistance in this pest [71]. Similarly, in *T*. *cinnabarinus*, a lncRNA acts as a sponge for a miRNA and reduces its binding to a GST-encoding mRNA, ultimately leading to increased GST protein levels and cyflumetofen resistance [43]. Furthermore, using RNAi of lncRNA genes, a study by Peng et al., 2022 demonstrated a role for a lncRNA, MSTRG.36649.2/5, in positive regulation of *CYP6CY21* in cotton aphid *Aphis gossypii* G., resulting in spirotetramat resistance [36]. Interestingly, the same study also found that another lncRNA, MSTRG.71880.1, negatively regulates *CYP380C6* expression, which also contributes to spirotetramat resistance in this pest. However, the mechanism by which these two lncRNAs regulated the CYP genes in *A*. *gossypii* was not clear, as there was no evidence of an miRNA sponge effect. In our *in silico* analysis, we identified four binding sites for the *lncRNA-2* on *CYP9E2* mRNA. Additionally, knock-down of *lncRNA-2* resulted in a significant decrease in *CYP9E2* levels. These findings suggest that the *CYP9E2* gene is positively regulated by the *lncRNA-2* in CPB, which in turn contributes to spinosad resistance. There are multiple ways by which lncRNA could regulate gene expression, such as directly binding to target mRNA to stabilize its levels or acting as precursor for miRNA production or miRNA sponges [40, 41]. Based on our results, we suggest that *CYP9E2* regulation might be accomplished by interaction of *lncRNA-2* with the *CYP9E2* mRNA, since downregulation of the former results in downregulation of the latter, and *in silico* analysis supports this hypothesis. However, regulation could also be through abovementioned mechanisms such as miRNA sponge effects; therefore, future studies could focus on identifying the exact mechanism by which *lncRNA-2* regulates *CYP9E2* expression.

Given that the simultaneous knock-down of *lncRNA-2* and *CYP9E2* genes did not completely eliminate spinosad resistance, and multiple miRNAs and CYPs were upregulated in CPB upon spinosad exposure [29, 30], we cannot overlook the potential role of miRNAs and other resistance-related genes in spinosad resistance in CPB. For instance, *CYP6A23* and *CYP4G15* levels were both upregulated upon exposure to low levels of spinosad in another CPB population, and subsequent bioassays using RNAi and spinosad exposure demonstrated a partial, albeit non-significant, role of *CYP4G15*, but not *CYP6A23*, in resistance [29]. Interestingly, our results also identified *CYP6A23* as constitutively upregulated in our OFP population based on RNA-seq results; however, as this gene was already shown to not play a role in spinosad resistance, it was not pursued here.

In summary, results from our study provide strong evidence that metabolic resistance mediated by CYP enzymes plays a role in resistance to spinosad in CPB. The constitutive overexpression of *CYP9E2* and its positive regulator, *lncRNA-2*, presumably allows resistant beetles to produce elevated levels of this enzyme, which in return allows enhanced metabolism of spinosad molecules, leading to resistance. However, our results also imply that, in CPB, resistance to spinosad is polygenic, and more studies are needed to identify other genes, as well as the potential roles of miRNAs in resistance. Ultimately, the knowledge gained from this study could help develop efficient control strategies of CPB by utilizing RNAi of resistance related genes in combination with spinosad, so that these naturally-derived insecticides can be employed for pest control for years to come.

## Supporting information

S1 Table Entrust dilutions used for insecticide bioassays. (DOCX)

S2 Table List of primers used in this study. (DOCX)

S3 Table Summary of RNA-seq data before and after mapping. (DOCX)

S4 Table List of differentially expressed transcripts in OFP compared with CFP of CPB. (DOCX)

S5 Table Fold change differences in RNA-seq and RT-qPCR data in OFP. (DOCX)

S6 Table Interaction of *lncRNA-2* with *CYP9E2* mRNA as predicted by IntaRNAv2 software. (DOCX)

S1 Fig Confirmation of dsRNA production in *E*. *coli* HT1115 cells. (DOCX)

S2 Fig Volcano plot showing differentially expressed transcripts between CFP and OFP of CPB. (DOCX)

S3 Fig Molecular pathway enrichment analyses of differentially expressed transcripts between two field populations of CPB. A) molecular pathways enriched in OFP; B) molecular pathways enriched in CFP.
(DOCX)
