# Supplementary material for: Role of CYP9E2 and a long non-coding RNA gene in resistance to a spinosad insecticide in the Colorado potato beetle, Leptinotarsa decemlineata
Source: PLoS One. 2024 May 24;19(5):e0304037. doi: 10.1371/journal.pone.0304037 (PMC11125468; doi:10.1371/journal.pone.0304037)
Supplement: S1 Table — (DOCX) [file pone.0304037.s001.docx]

**S1 Table.** **Entrust dilutions used for insecticide bioassays.**

| Sensitive laboratory population (SLP) | | Conventional Farm  (CFP) | | Organic Farm  (OFP) | |
| --- | --- | --- | --- | --- | --- |
| Dose (ppm) | **# of CPB used** | **Dose (ppm)** | **# of CPB used** | **Dose (ppm)** | **# of CPB used** |
| 0 | 32 | 0 | 33 | 0 | 33 |
| 100 | 30 | 250 | 33 | 500 | 31 |
| 250 | 29 | 500 | 31 | 750 | 29 |
| 500 | 31 | 1000 | 33 | 900 | 31 |
| 750 | 30 | 1500 | 34 | 1000 | 30 |
| 900 | 31 | 2000 | 31 | 1500 | 30 |
| 1000 | 31 | 3000 | 31 | 2000 | 31 |
| 1500 | 31 | 4000 | 30 | 3000 | 31 |
|  |  | 5000 | 33 | 4000 | 31 |
|  |  |  |  | 5000 | 30 |
|  |  |  |  | 6000 | 35 |
|  |  |  |  | 8000 | 32 |
|  |  |  |  | 10000 | 31 |
|  |  |  |  | 12000 | 35 |
|  |  |  |  | 14000 | 36 |
