# Supplementary material for: Role of CYP9E2 and a long non-coding RNA gene in resistance to a spinosad insecticide in the Colorado potato beetle, Leptinotarsa decemlineata
Source: PLoS One. 2024 May 24;19(5):e0304037. doi: 10.1371/journal.pone.0304037 (PMC11125468; doi:10.1371/journal.pone.0304037)
Supplement: S2 Table — (DOCX) [file pone.0304037.s002.docx]

**S2 Table. List of primers used in this study.**

| **Target Gene** | **Forward and reverse primers**  **(5’-3’)^1^** | **Primer**  **Efficiency (%)** | **Amplicon**  **size (bp)** |
| --- | --- | --- | --- |
| **Primers used in RT-qPCR** | | | |
| *CYP9E2* | TATGCGCCGTTTGGATTTGG  GCTCTCAAAGGTGTGCAAGTTT | 93.3 | 137 |
| *lncRNA-1* | CGGCCTTAACTCCATCACTCA  TGAAACGAGACCTTCTGGCG | 92.1 | 141 |
| *lncRNA-2* | TTGGAAGGATTGCAAGTACAGGA  GCAGTCGCATCTTCCATTGAC | 91.9 | 123 |
| *CYP6A13* | GGAGTTGCACATCTGGTTAAGAA  GACCTCCAACAGCAGAGATGA | 91.9 | 106 |
| *CYP6A23* | AGCGCAGTGTCTCGTGTTCT  TCCTGGTTGAGGGACAGTTCA | 96.3 | 90 |
| *L8E* | GGTAACCATCAACACATTGG  TCTTGGCATCCACTTTACC | 97.4 | 124 |
| *ARF1* | GACTGCAAGTAGGAGAAGTTG  TCGGCAGAGTCTACCACAT | 94.1 | 181 |
| *EF1Α* | CAGGGCAAGGTTTGAAAGATAA  CCATCAGCACAGTTCCCAT | 99.6 | 168 |
| **Primers used in cloning of dsRNA fragments for RNA interference** | | | |
| *CYP9E2* | TAGCGGCCGCCGCAGGCATTGATTTTCTTT  ACAGGTCGACTTTCTGGATCAAAACGGTCC | - | 388 |
| *lncRNA-2* | TAGCGGCCGCCAGCCATCAATTCTTCTCCAG  ACAGGTCGACCCATTGACGCATTTGTAA | - | 146 |
| *GFP* | TAGCGGCCGCCCATGCCCGAAGGTTATGTA  ACAGGTCGACGGACAGGTAATGGTTGTCTGG | - | 449 |

^1^*Not*I and *Sal*I restriction enzyme cut sites are underlined in primers used for cloning.
