# Supplementary material for: Role of CYP9E2 and a long non-coding RNA gene in resistance to a spinosad insecticide in the Colorado potato beetle, Leptinotarsa decemlineata
Source: PLoS One. 2024 May 24;19(5):e0304037. doi: 10.1371/journal.pone.0304037 (PMC11125468; doi:10.1371/journal.pone.0304037)
Supplement: S3 Table — (DOCX) [file pone.0304037.s003.docx]

**S3 Table. Summary of RNA-seq data before and after mapping.**

| Sample | Raw reads | Reads remaining  after QC | Reads mapped in pairs (%) | Reads mapped in broken pairs (%)^*^ |
| --- | --- | --- | --- | --- |
| CFP bio1 | 146,261,028 | 145,911,194 | 59.06 | 7.89 |
| CFP bio2 | 151,124,270 | 150,805,088 | 59.04 | 8.14 |
| CFP bio3 | 149,537,850 | 149,262,368 | 59.13 | 7.83 |
| OFP bio1 | 167,095,940 | 166,722,376 | 58.72 | 8.08 |
| OFP bio2 | 183,509,980 | 183,162,652 | 59.38 | 8.35 |
| OFP bio3 | 144,953,604 | 144,686,456 | 58.72 | 8.08 |

* A pair was marked broken, either because only one read in the pair mapped, or because the distance or relative orientation was wrong.
