# Supplementary material for: Role of CYP9E2 and a long non-coding RNA gene in resistance to a spinosad insecticide in the Colorado potato beetle, Leptinotarsa decemlineata
Source: PLoS One. 2024 May 24;19(5):e0304037. doi: 10.1371/journal.pone.0304037 (PMC11125468; doi:10.1371/journal.pone.0304037)
Supplement: S4 Table — (DOCX) [file pone.0304037.s004.docx]

**S4 Table. List of differentially expressed transcripts in OFP compared with CFP of CPB.**

| **Transcript name^1^** | **Fold change** | **FDR *P*-value** | **Sequence description** | **Regulation** |
| --- | --- | --- | --- | --- |
| LDEC006701-RA | 1055.196 | 0.017365 | three-prime repair exonuclease 1-like | Up |
| LDEC022920-RA | 812.1394 | 0.019451 | transmembrane protease serine 9-like | Up |
| LDEC017050-RA | 141.4109 | 1.32E-07 | zinc finger BED domain-containing protein 5-like | Up |
| LDEC022764-RA | 107.2849 | 0 | cathepsin B-like isoform X2 | Up |
| LDEC024713-RA | 91.45136 | 5.00E-06 | 26S proteasome non-ATPase regulatory subunit 1-like | Up |
| LDEC003898-RA | 67.92514 | 4.41E-06 | uncharacterized protein LOC111509689 | Up |
| LDEC012879-RA | 44.15858 | 0.034826 | uncharacterized protein LOC111506598 | Up |
| LDEC018918-RA | 30.47128 | 0.044698 | uncharacterized protein LOC111511026 | Up |
| LDEC023055-RA | 30.387 | 2.70E-03 | zinc finger MYM-type protein 1-like | Up |
| LDEC000820-RA | 27.3523 | 6.08E-03 | piggyBac transposable element-derived protein 4-like | Up |
| LDEC003372-RA | 24.64902 | 8.48E-13 | transmembrane protein 145-like | Up |
| LDEC004496-RA | 20.76633 | 2.68E-03 | probable splicing factor, arginine/serine-rich 6 | Up |
| LDEC019791-RA | 20.60934 | 8.06E-03 | uncharacterized protein LOC111503745 | Up |
| LDEC022482-RA | 18.91101 | 4.04E-05 | uncharacterized protein LOC111515969 | Up |
| LDEC005250-RA | 18.71598 | 4.03E-08 | crustapain-like isoform X2 | Up |
| LDEC022045-RA | 18.70995 | 0.028011 | gamma-glutamylcyclotransferase-like isoform X1 | Up |
| LDEC016460-RA | 17.53484 | 0.035248 | uncharacterized protein LOC111508338 | Up |
| LDEC023800-RA | 17.08042 | 1.96E-03 | golgin subfamily A member 6-like protein 6 | Up |
| LDEC018503-RA | 16.70712 | 4.47E-05 | mental retardation GTPase activating protein homolog 4-like, partial | Up |
| LDEC011597-RA | 16.22754 | 0.036851 | uncharacterized protein LOC111510519 | Up |
| LDEC019894-RA | 15.69149 | 4.84E-03 | uncharacterized protein LOC111510050 | Up |
| LDEC017036-RA | 14.89585 | 4.00E-07 | pickpocket protein 28-like | Up |
| LDEC008714-RA | 14.84304 | 7.13E-04 | cuticular protein 44-aa motif 1 | Up |
| LDEC018834-RA | 14.53191 | 8.06E-03 | uncharacterized protein LOC111516278 | Up |
| LDEC009727-RA | 13.96301 | 5.57E-07 | piggyBac transposable element-derived protein 3-like | UP |
| LDEC019936-RA | 13.59849 | 3.52E-08 | uncharacterized protein LOC111516266 | Up |
| LDEC003227-RA | 12.73642 | 0.02671 | KRAB-A domain-containing protein 2-like | Up |
| LDEC006956-RA | 12.669 | 1.44E-03 | kynurenine 3-monooxygenase-like | Up |
| LDEC001829-RA | 12.52355 | 1.93E-05 | endocuticle structural glycoprotein ABD-4-like | Up |
| LDEC000698-RA | 12.3768 | 3.38E-03 | kunitz-type serine protease inhibitor HCRG1-like | Up |
| LDEC010956-RA | 12.01083 | 2.05E-03 | uncharacterized protein LOC111513268 | Up |
| LDEC015059-RA | 11.12183 | 7.08E-03 | clock | Up |
| LDEC016824-RA | 10.92365 | 6.51E-05 | uncharacterized protein LOC111512998 | Up |
| LDEC019628-RB | 10.77735 | 0.024443 | uncharacterized protein LOC111515158 | Up |
| LDEC022112-RA | 10.12822 | 0.024617 | uncharacterized protein LOC111516621 | Up |
| LDEC005296-RA | 10.0895 | 2.78E-04 | zinc finger BED domain-containing protein 5-like | Up |
| LDEC005974-RA | 10.03381 | 0.017294 | serine proteinase stubble-like | Up |
| LDEC021147-RA | 9.475466 | 3.43E-03 | golgin subfamily A member 6-like protein 6 | Up |
| LDEC021385-RA | 9.116086 | 3.24E-03 | mental retardation GTPase activating protein homolog 4-like, partial | Up |
| LDEC022017-RA | 9.081258 | 0.035248 | uncharacterized protein LOC111514644 | Up |
| LDEC013538-RA | 8.450018 | 5.93E-06 | probable cytochrome P450 6a23 | Up |
| LDEC003725-RA | 8.361749 | 1.50E-03 | uncharacterized protein LOC111508042 | Up |
| LDEC014173-RA | 7.808072 | 0.010756 | probable multidrug resistance-associated protein lethal(2)03659 | Up |
| LDEC019566-RA | 7.807481 | 3.40E-03 | catalase-like | Up |
| LDEC011084-RA | 7.701027 | 0.026212 | protein yellow-like | Up |
| LDEC010306-RA | 7.70006 | 1.23E-03 | actin cytoskeleton-regulatory complex protein PAN1-like | Up |
| LDEC001711-RA | 7.535717 | 0.024186 | myrosinase 1-like | Up |
| LDEC008589-RA | 7.475165 | 1.78E-03 | macrophage mannose receptor 1-like | Up |
| LDEC024730-RA | 7.408862 | 0.02646 | putative nuclease HARBI1 | Up |
| LDEC008202-RA | 7.401734 | 1.69E-04 | transcription factor Sp9-like | Up |
| LDEC021826-RA | 7.397676 | 0.024443 | PREDICTED: Leptinotarsa decemlineata uncharacterized LOC111516474 (LOC111516474), ncRNA | Up |
| LDEC017109-RA | 7.334726 | 0.040218 | uncharacterized protein LOC111516352 | Up |
| LDEC010104-RA | 7.216786 | 0.04181 | uncharacterized protein LOC111513890 | Up |
| LDEC001834-RA | 7.1116 | 0.024271 | endocuticle structural glycoprotein ABD-4-like | Up |
| LDEC023561-RA | 7.019145 | 0.024355 | uncharacterized protein LOC111514644 | Up |
| LDEC021753-RA | 6.9827 | 0.013761 | ankyrin-3 isoform X1 | Up |
| LDEC005603-RA | 6.969128 | 3.43E-03 | intraflagellar transport protein 46 homolog | Up |
| LDEC020236-RA | 6.945272 | 4.90E-03 | uncharacterized protein LOC111508515 isoform X2 | Up |
| LDEC005867-RA | 6.821269 | 8.06E-03 | ionotropic receptor 25a | Up |
| LDEC009396-RA | 6.712206 | 2.31E-03 | uncharacterized protein LOC111502312 | Up |
| LDEC009031-RA | 6.586022 | 7.28E-04 | osmotic avoidance abnormal protein 3-like isoform X4 | Up |
| LDEC008080-RA | 6.563406 | 4.84E-03 | myb-like protein X | Up |
| LDEC018683-RA | 6.528849 | 0.023367 | guanylate cyclase 32E-like, partial | Up |
| LDEC024514-RA | 6.231317188 | 4.84E-03 | unknown | Up |
| LDEC018001-RA | 6.093638 | 0.028184 | uncharacterized protein LOC111514694 | Up |
| LDEC022098-RA | 6.087276 | 0.012365 | headcase protein-like | Up |
| LDEC018084-RA | 6.070758 | 8.06E-03 | putative uncharacterized protein DDB_G0282133, partial | Up |
| LDEC009584-RA | 6.055439 | 2.96E-03 | chitinase 3-like | Up |
| LDEC010305-RA | 5.842587 | 7.04E-03 | actin cytoskeleton-regulatory complex protein PAN1-like | Up |
| LDEC017924-RA | 5.835732 | 0.010997 | neuroligin-1-like | Up |
| LDEC018682-RA | 5.670221 | 6.27E-03 | guanylate cyclase 32E-like, partial | Up |
| LDEC005642-RA | 5.556398 | 0.035327 | Down syndrome cell adhesion molecule-like protein Dscam2 | Up |
| LDEC008081-RA | 5.421507 | 0.024186 | uncharacterized protein LOC111505396 | Up |
| LDEC013883-RA | 5.388973 | 0.024355 | laccase-like | Up |
| LDEC019178-RA | 5.225786 | 6.30E-03 | probable tubulin polyglutamylase TTLL2 isoform X2 | Up |
| LDEC021953-RA | 5.166394 | 0.040218 | cuticular protein 42 | Up |
| LDEC013973-RA | 5.147273 | 0.014 | pancreatic triacylglycerol lipase-like | Up |
| LDEC018796-RA | 5.124197 | 0.027338 | protein antagonist of like heterochromatin protein 1-like | Up |
| LDEC019331-RA | 5.03076 | 0.022311 | uncharacterized protein LOC111512853 | Up |
| LDEC009209-RA | 4.996529 | 7.53E-03 | cathepsin B-like | Up |
| LDEC013407-RA | 4.975278 | 0.024355 | voltage-dependent calcium channel type A subunit alpha-1, partial | Up |
| LDEC021333-RA | 4.804157 | 9.43E-04 | cytochrome P450 9e2-like isoform X2 | Up |
| LDEC022309-RA | 4.802185 | 9.79E-03 | cytochrome P450 9e2-like isoform X2 | Up |
| LDEC012424-RA | 4.751086 | 3.24E-03 | neo-calmodulin-like isoform X2 | Up |
| LDEC016043-RA | 4.736114 | 3.40E-03 | uncharacterized protein LOC111502734 | Up |
| LDEC003579-RA | 4.672301 | 0.046444 | uncharacterized threonine-rich GPI-anchored glycoprotein PJ4664.02-like | Up |
| LDEC012483-RA | 4.631083 | 0.042816 | uncharacterized protein LOC111506478 | Up |
| LDEC005248-RA | 4.544197 | 0.020827 | crustapain-like isoform X1 | Up |
| LDEC014724-RA | 4.52116 | 2.34E-03 | protein toll-like | Up |
| LDEC017619-RA | 4.493739 | 0.029961 | uncharacterized protein LOC111505574 | Up |
| LDEC008839-RA | 4.428239 | 0.019688 | protein SCAI isoform X2 | Up |
| LDEC007240-RA | 4.389471 | 0.029878 | uncharacterized protein LOC111503828 | Up |
| LDEC005639-RA | 4.295322 | 0.019344 | Down syndrome cell adhesion molecule-like protein Dscam2 | Up |
| LDEC017037-RA | 4.230182 | 0.017845 | pickpocket protein 28-like | Up |
| LDEC002117-RA | 4.215739 | 0.030295 | putative nuclease HARBI1 | Up |
| LDEC006176-RA | 4.210522 | 0.019451 | serine/arginine-rich splicing factor RS2Z33-like | Up |
| LDEC005255-RA | 4.094966 | 0.030811 | crustapain-like isoform X1 | Up |
| LDEC018262-RA | 3.881103 | 0.035076 | glucose transporter type 1-like, partial | Up |
| LDEC022531-RA | 3.857387 | 0.016868 | uncharacterized protein LOC111513291 | Up |
| LDEC021334-RA | 3.787001 | 0.030811 | cytochrome P450 9e2-like isoform X2 | Up |
| LDEC011978-RA | -3.88074 | 0.026212 | 60S acidic ribosomal protein P0 | Down |
| LDEC014467-RA | -4.01572 | 0.013761 | cytochrome P450 6k1-like isoform X1 | Down |
| LDEC011277-RA | -4.05168 | 0.026212 | uncharacterized protein LOC111506853 | Down |
| LDEC009487-RA | -4.19927 | 0.024355 | uncharacterized protein LOC111512694 | Down |
| LDEC020177-RA | -4.28761 | 0.035886 | fibropellin-1-like | Down |
| LDEC002882-RA | -4.29845 | 0.041551 | uncharacterized protein LOC111501996 | Down |
| LDEC014464-RA | -4.39653 | 0.028011 | unknown | Down |
| LDEC023726-RA | -4.54583 | 0.030139 | cytochrome P450 412a1 | Down |
| LDEC020560-RA | -4.57513 | 0.023367 | juvenile hormone acid O-methyltransferase-like isoform X1 | Down |
| LDEC007118-RA | -4.57881 | 0.021494 | uncharacterized protein LOC111506618 | Down |
| LDEC007955-RA | -4.74182 | 0.024271 | alpha-tocopherol transfer protein-like | Down |
| LDEC004243-RA | -4.7918 | 8.06E-03 | cuticular protein 48 | Down |
| LDEC021941-RA | -4.9291 | 0.027338 | multidrug resistance-associated protein 1-like, partial | Down |
| LDEC019091-RA | -5.14442 | 7.95E-03 | PREDICTED: Leptinotarsa decemlineata uncharacterized LOC111514973 (LOC111514973), ncRNA | Down |
| LDEC005430-RA | -5.18401 | 0.044551 | lactase-phlorizin hydrolase-like | Down |
| LDEC007775-RA | -5.2353 | 3.93E-04 | golgin subfamily A member 6-like protein 22, partial | Down |
| LDEC021344-RA | -5.3953 | 0.034427 | uncharacterized protein LOC111513104 | Down |
| LDEC019211-RA | -5.60856 | 0.029961 | myrosinase 1-like | Down |
| LDEC004419-RA | -5.81252 | 6.70E-04 | uncharacterized protein LOC111501891 | Down |
| LDEC017770-RA | -5.97296 | 0.029234 | apyrase-like isoform X1 | Down |
| LDEC022355-RA | -6.10938 | 0.03542 | uncharacterized protein LOC111515178 | Down |
| LDEC009324-RA | -6.11696 | 3.78E-04 | uncharacterized protein LOC111505660 | Down |
| LDEC018501-RA | -6.14617 | 0.013415 | putative nuclease HARBI1 | Down |
| LDEC021819-RA | -6.29889 | 2.57E-04 | glucose dehydrogenase [FAD, quinone]-like | Down |
| LDEC024211-RA | -6.38623 | 0.025599 | zinc finger BED domain-containing protein 5-like | Down |
| LDEC001258-RA | -6.47256 | 4.06E-03 | uncharacterized protein LOC111512091 | Down |
| LDEC000759-RA | -6.47495 | 3.30E-04 | tektin-3-like | Down |
| LDEC002555-RA | -6.52453 | 4.27E-03 | TBC1 domain family member 5 homolog A-like | Down |
| LDEC018732-RA | -6.53749 | 0.030811 | guanine nucleotide exchange factor MSS4 homolog | Down |
| LDEC006289-RA | -6.59433 | 0.026985 | PREDICTED: Leptinotarsa decemlineata glycine-rich cell wall structural protein 1-like (LOC111510965), transcript variant X1, mRNA | Down |
| LDEC001184-RA | -6.79377 | 1.78E-03 | uncharacterized protein LOC111502381 | Down |
| LDEC020006-RA | -6.85564 | 1.35E-03 | blood vessel epicardial substance-A-like | Down |
| LDEC018444-RA | -6.87528 | 1.41E-03 | PREDICTED: Leptinotarsa decemlineata cubilin-like (LOC111505439), mRNA | Down |
| LDEC014468-RA | -6.90492 | 3.93E-04 | cytochrome P450 6k1-like isoform X2 | Down |
| LDEC011760-RA | -7.25295 | 0.030908 | No match | Down |
| LDEC006290-RA | -7.31644 | 8.06E-03 | glycine-rich cell wall structural protein 1.8-like isoform X3 | Down |
| LDEC007721-RA | -7.40293 | 0.01928 | piggyBac transposable element-derived protein 4-like | Down |
| LDEC023926-RA | -7.46422 | 3.25E-03 | glucose dehydrogenase [FAD, quinone]-like | Down |
| LDEC016213-RA | -7.69206 | 3.52E-03 | chromo domain-containing protein cec-1-like | Down |
| LDEC014023-RA | -7.84404 | 0.019451 | uncharacterized protein LOC111509369 | Down |
| LDEC004414-RA | -7.87307 | 4.90E-03 | cuticular protein tweedle motif 8 | Down |
| LDEC016610-RA | -8.13406 | 0.046523 | uncharacterized protein LOC111514381 [ | Down |
| LDEC017615-RA | -8.13948 | 2.96E-04 | peroxisomal hydratase-dehydrogenase-epimerase-like | Down |
| LDEC017543-RA | -8.31455 | 2.32E-03 | PREDICTED: LOW QUALITY PROTEIN: diuretic hormone receptor-like | Down |
| LDEC004225-RA | -8.50522 | 0.046523 | PREDICTED: Leptinotarsa decemlineata uncharacterized LOC111504060 (LOC111504060), ncRNA | Down |
| LDEC011571-RA | -8.55514 | 1.84E-04 | trypsin-like | Down |
| LDEC023568-RA | -8.5965 | 5.40E-04 | No match | Down |
| LDEC014830-RA | -8.64243 | 3.56E-05 | No match | Down |
| LDEC007026-RA | -8.85279 | 5.86E-04 | uncharacterized protein LOC111503383 | Down |
| LDEC021907-RA | -8.94926 | 0.042134 | uncharacterized protein LOC111503708 | Down |
| LDEC015661-RA | -9.08948 | 0.024443 | 40S ribosomal protein S7 | Down |
| LDEC020876-RA | -9.25986 | 1.69E-04 | No match | Down |
| LDEC008672-RA | -9.2721 | 0.024355 | LOW QUALITY PROTEIN: uncharacterized protein LOC111507449 [ | Down |
| LDEC022848-RA | -9.59088 | 1.44E-03 | unnamed protein product, partial | Down |
| LDEC006844-RA | -9.74136 | 0.022311 | octopamine receptor beta-3R-like | Down |
| LDEC007904-RA | -9.77857 | 2.74E-05 | kinesin-like protein KIF19 | Down |
| LDEC017193-RA | -10.2633 | 0.028184 | nuclear apoptosis-inducing factor 1-like | Down |
| LDEC007176-RA | -10.2858 | 4.63E-06 | zinc finger protein 271-like | Down |
| LDEC013042-RA | -10.3117 | 0.046825 | dolichyl-diphosphooligosaccharide--protein glycosyltransferase subunit 1 | Down |
| LDEC021645-RA | -10.3262 | 8.36E-05 | peroxidase-like | Down |
| LDEC002474-RA | -10.4407 | 1.78E-03 | PREDICTED: Leptinotarsa decemlineata uncharacterized LOC111511088 (LOC111511088), ncRNA | Down |
| LDEC016945-RA | -10.6639 | 2.96E-04 | glucose dehydrogenase [FAD, quinone]-like, partial [ | Down |
| LDEC022849-RA | -10.6924 | 0.026718 | uncharacterized protein LOC111513006 | Down |
| LDEC018656-RA | -10.8944 | 0.044446 | uncharacterized protein LOC111510575 | Down |
| LDEC008711-RA | -10.9518 | 1.93E-05 | cuticle protein LPCP-23-like | Down |
| LDEC012010-RA | -10.9783 | 7.22E-05 | piggyBac transposable element-derived protein 2-like | Down |
| LDEC019706-RA | -11.024 | 9.35E-04 | stress response protein NST1-like | Down |
| LDEC009115-RA | -11.066 | 0.04334 | MAGE-like protein 2 | Down |
| LDEC000590-RA | -11.4369 | 3.00E-05 | nose resistant to fluoxetine protein 6-like | Down |
| LDEC016632-RA | -11.6653 | 1.81E-03 | cuticular protein analogous to peritrophins 1-L | Down |
| LDEC023477-RA | -11.9764 | 3.56E-05 | glucose dehydrogenase [FAD, quinone]-like | Down |
| LDEC019213-RA | -12.0723 | 1.44E-04 | myrosinase 1-like | Down |
| LDEC016946-RA | -12.2067 | 0.013309 | glucose dehydrogenase [FAD, quinone]-like | Down |
| LDEC020810-RA | -12.3435 | 6.75E-04 | putative nuclease HARBI1 | Down |
| LDEC020683-RA | -12.763 | 1.78E-03 | putative nuclease HARBI1 | Down |
| LDEC022562-RA | -13.0545 | 1.91E-07 | reticulocyte-binding protein 2 homolog a-like | Down |
| LDEC021067-RA | -13.4692 | 6.80E-03 | THAP domain-containing protein 2-like | Down |
| LDEC012313-RA | -13.8426 | 0.024443 | No match | Down |
| LDEC009472-RA | -14.0089 | 8.33E-04 | uncharacterized protein LOC111502858 | Down |
| LDEC000117-RA | -14.4503 | 1.24E-04 | uncharacterized protein LOC111503967 | Down |
| LDEC010847-RA | -14.4782 | 4.84E-03 | cuticular protein 50 | Down |
| LDEC021674-RA | -16.2429 | 3.24E-03 | uncharacterized protein LOC111516400 | Down |
| LDEC007996-RA | -16.2859 | 1.18E-04 | uncharacterized protein LOC111504870 | Down |
| LDEC016067-RA | -17.8162 | 1.85E-08 | uncharacterized protein LOC111506939 | Down |
| LDEC016635-RA | -18.3808 | 4.03E-08 | vitellogenin-like | Down |
| LDEC018113-RA | -21.1002 | 1.08E-05 | uncharacterized protein LOC111510934 | Down |
| LDEC003196-RA | -21.3501 | 0.032392 | uncharacterized protein LOC111502963 [Leptinotarsa decemlineata] | Down |
| LDEC005731-RA | -21.5348 | 5.57E-07 | equilibrative nucleoside transporter 3-like isoform X2 | Down |
| LDEC008710-RA | -22.0557 | 2.74E-05 | cuticle protein LPCP-23-like | Down |
| LDEC002701-RA | -22.8955 | 5.72E-12 | uncharacterized protein LOC111514409 | Down |
| LDEC011029-RA | -24.5322 | 4.18E-06 | vitellogenin-like | Down |
| LDEC013950-RA | -25.1192 | 1.69E-04 | uncharacterized protein LOC111510473 | Down |
| LDEC020760-RA | -26.7163 | 1.54E-09 | E3 ubiquitin-protein ligase Siah1-like | Down |
| LDEC008662-RA | -31.661 | 1.97E-03 | uncharacterized protein LOC111516951 | Down |
| LDEC008588-RA | -44.021 | 0.011356 | macrophage mannose receptor 1-like | Down |
| LDEC011343-RA | -65.0682 | 0 | probable cytochrome P450 6a13 | Down |
| LDEC003197-RA | -258.201 | 2.90E-06 | uncharacterized protein LOC111502974 [Leptinotarsa decemlineata] | Down |
| LDEC021505-RA | -700.814 | 0.012853 | ATP-dependent RNA helicase WM6-like | Down |
| LDEC016640-RA | -1410.14 | 3.46E-11 | trypsin-3-like | Down |

^1^Transcript name is from lepdec_OGSv1.1 transcriptome downloaded from <https://data.nal.usda.gov/dataset/leptinotarsa-decemlineata-official-gene-set-v11>)
