# Supplementary material for: Role of CYP9E2 and a long non-coding RNA gene in resistance to a spinosad insecticide in the Colorado potato beetle, Leptinotarsa decemlineata
Source: PLoS One. 2024 May 24;19(5):e0304037. doi: 10.1371/journal.pone.0304037 (PMC11125468; doi:10.1371/journal.pone.0304037)
Supplement: S5 Table — (DOCX) [file pone.0304037.s005.docx]

**S5 Table. Fold change differences in RNA-seq and RT-qPCR data in OFP.**

| Transcript name^1^ | **Gene** | **Fold change in RNA-seq** | **Fold change in RT-qPCR** |
| --- | --- | --- | --- |
| LDEC021826-RA | *lncRNA-1* | 7.4 | 1.2 |
| LDEC021333-RA | *CYP9E2* | 4.80 | 1.71 |
| LDEC022309-RA | *CYP9E2* | 4.80 | 1.71 |
| LDEC021334-RA | *CYP9E2* | 3.79 | 1.71 |
| LDEC013538-RA | *CYP6A23* | 8.5 | 1.39 |
| LDEC004449-RA | *lncRNA-2* | Not applicable | 6.90 |
|  |  |  |  |

^1^Transcript name is from lepdec_OGSv1.1 transcriptome downloaded from <https://data.nal.usda.gov/dataset/leptinotarsa-decemlineata-official-gene-set-v11>)
