# Supplementary material for: Role of CYP9E2 and a long non-coding RNA gene in resistance to a spinosad insecticide in the Colorado potato beetle, Leptinotarsa decemlineata
Source: PLoS One. 2024 May 24;19(5):e0304037. doi: 10.1371/journal.pone.0304037 (PMC11125468; doi:10.1371/journal.pone.0304037)
Supplement: S6 Table — (DOCX) [file pone.0304037.s006.docx]

**S6 Table. Potential interaction of *lncRNA-2* with *CYP9E2* mRNA as predicted by IntaRNAv2 software.**

| tar[get](http://rna.informatik.uni-freiburg.de/IntaRNA/Result.jsp?t_top100_row=0&t_top100_rows=10&t_top100_srt=id1&toolName=IntaRNA&jobID=2152762) | [Start(T)](http://rna.informatik.uni-freiburg.de/IntaRNA/Result.jsp?t_top100_row=0&t_top100_rows=10&t_top100_srt=start1&toolName=IntaRNA&jobID=2152762) | [End(T)](http://rna.informatik.uni-freiburg.de/IntaRNA/Result.jsp?t_top100_row=0&t_top100_rows=10&t_top100_srt=end1&toolName=IntaRNA&jobID=2152762) | [Query](http://rna.informatik.uni-freiburg.de/IntaRNA/Result.jsp?t_top100_row=0&t_top100_rows=10&t_top100_srt=id2&toolName=IntaRNA&jobID=2152762) | [Start(Q)](http://rna.informatik.uni-freiburg.de/IntaRNA/Result.jsp?t_top100_row=0&t_top100_rows=10&t_top100_srt=start2&toolName=IntaRNA&jobID=2152762) | [End(Q)](http://rna.informatik.uni-freiburg.de/IntaRNA/Result.jsp?t_top100_row=0&t_top100_rows=10&t_top100_srt=end2&toolName=IntaRNA&jobID=2152762) | [Energy](http://rna.informatik.uni-freiburg.de/IntaRNA/Result.jsp?t_top100_row=0&t_top100_rows=10&t_top100_srt=E&toolName=IntaRNA&jobID=2152762) |
| --- | --- | --- | --- | --- | --- | --- |
| CYP9e2 | 1373 | 1426 | lncRNA | 149 | 192 | -13.35 |
| CYP9e2 | 1532 | 1548 | lncRNA | 250 | 265 | -6.91 |
| CYP9e2 | 1598 | 1605 | lncRNA | 278 | 285 | -4.11 |
| CYP9e2 | 1572 | 1578 | lncRNA | 342 | 348 | -1.63 |
|  |  |  |  |  |  |  |
