# Supplementary material for: Role of CYP9E2 and a long non-coding RNA gene in resistance to a spinosad insecticide in the Colorado potato beetle, Leptinotarsa decemlineata
Source: PLoS One. 2024 May 24;19(5):e0304037. doi: 10.1371/journal.pone.0304037 (PMC11125468; doi:10.1371/journal.pone.0304037)
Supplement: S1 Fig — (DOCX) [file pone.0304037.s007.docx]

**1 2 3 4**


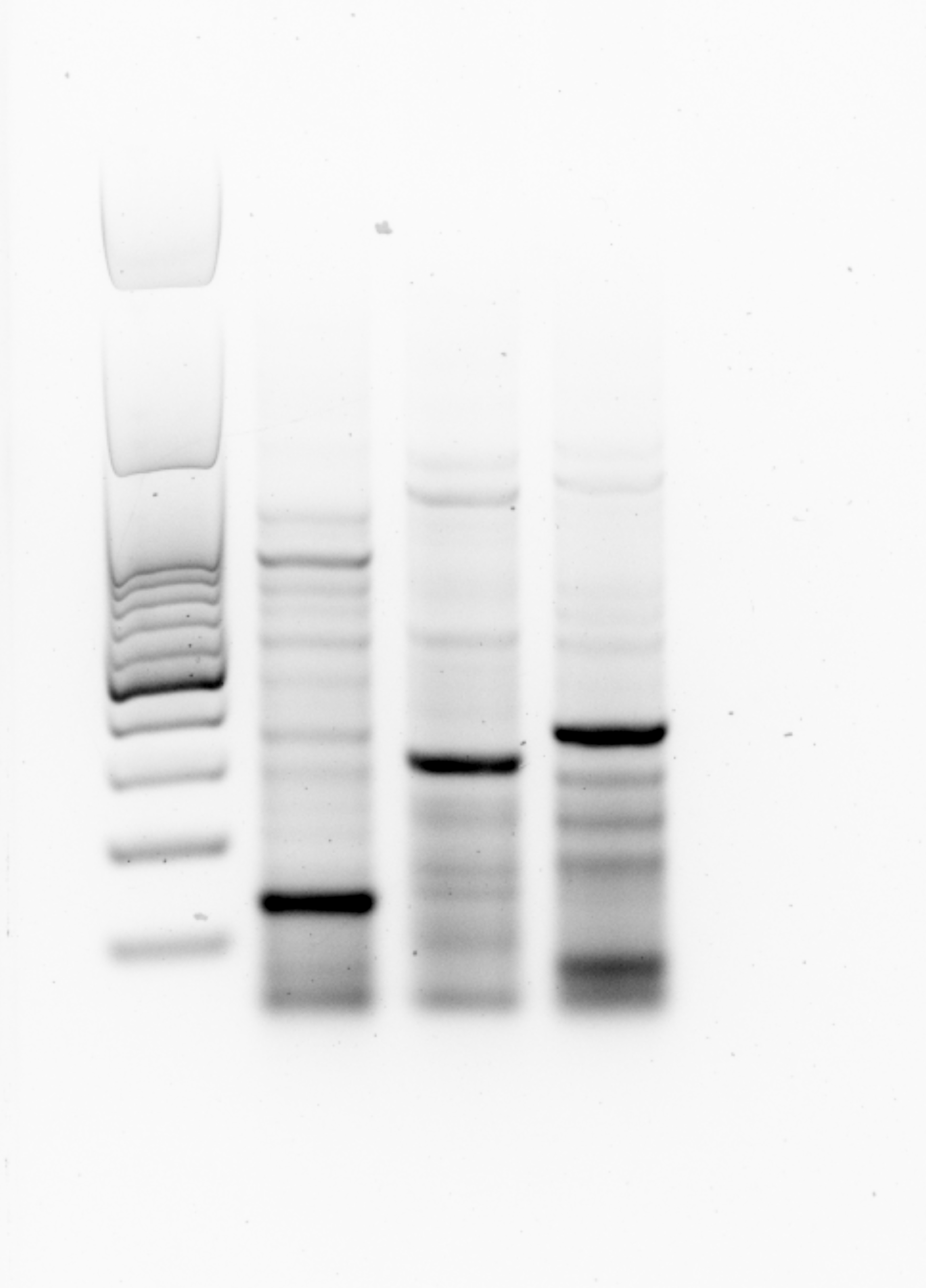


**S1 Fig. Confirmation of dsRNA production in *E. coli* HT1115 cells.** Red arrows point to the dsRNA molecules. 1 = 100 bp DNA ladder; 2 = *lncRNA-2* dsRNA; 3 = *CYP9E2* dsRNA; 4 = *GFP* dsRNA.
