# Supplementary material for: Role of CYP9E2 and a long non-coding RNA gene in resistance to a spinosad insecticide in the Colorado potato beetle, Leptinotarsa decemlineata
Source: PLoS One. 2024 May 24;19(5):e0304037. doi: 10.1371/journal.pone.0304037 (PMC11125468; doi:10.1371/journal.pone.0304037)
Supplement: S2 Fig — (DOCX) [file pone.0304037.s008.docx]

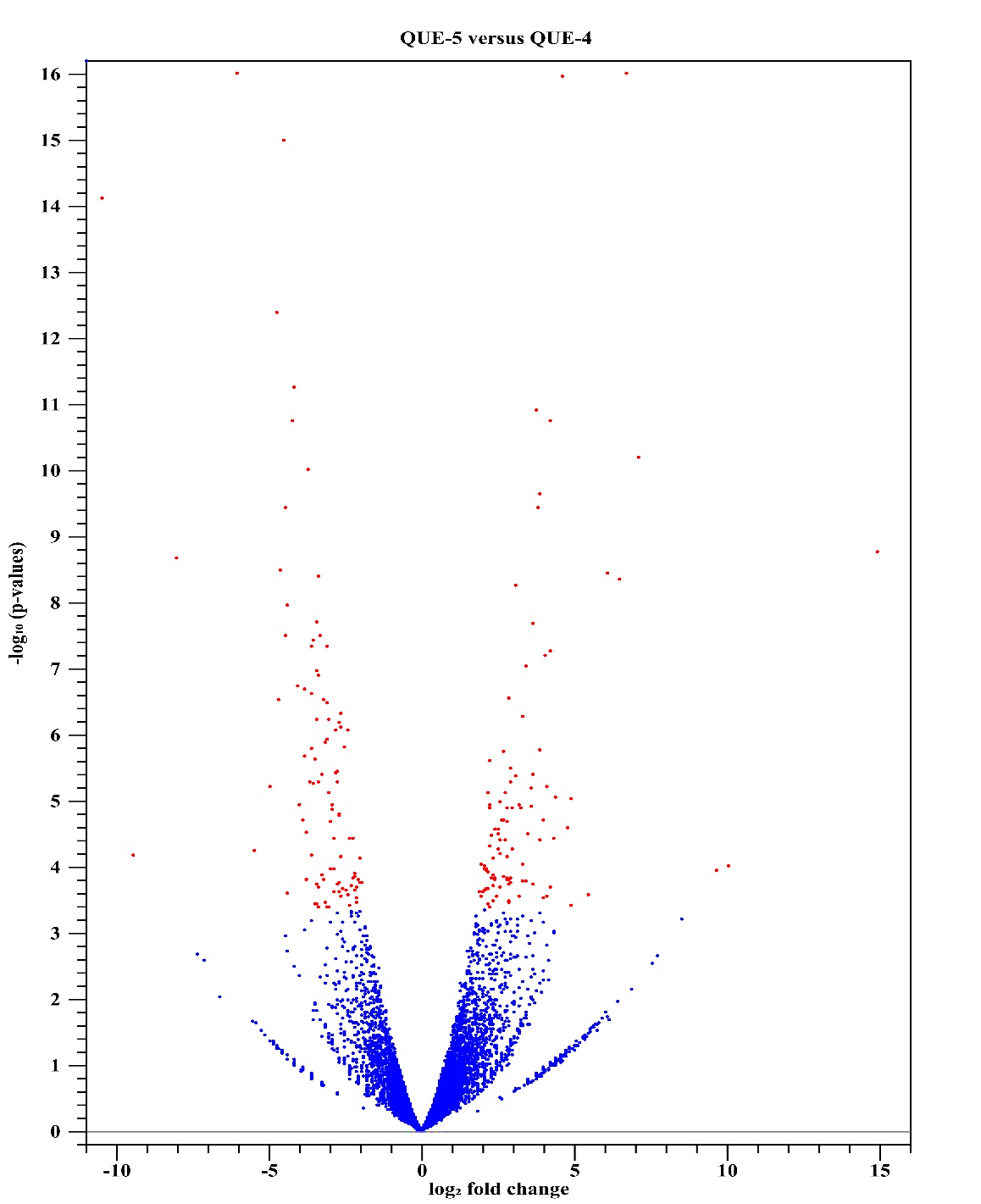


**S2 Fig.** **Volcano plot showing differentially expressed transcripts between CFP and OFP of CPB**. Each dot represents a transcript, and those that are differentially expressed at FDR of ≤ 0.05 and fold change of│log2│≥ 1 are coloured red.
