# Supplementary material for: Role of CYP9E2 and a long non-coding RNA gene in resistance to a spinosad insecticide in the Colorado potato beetle, Leptinotarsa decemlineata
Source: PLoS One. 2024 May 24;19(5):e0304037. doi: 10.1371/journal.pone.0304037 (PMC11125468; doi:10.1371/journal.pone.0304037)
Supplement: S3 Fig — A) molecular pathways enriched in OFP; B) molecular pathways enriched in CFP. (DOCX) [file pone.0304037.s009.docx]

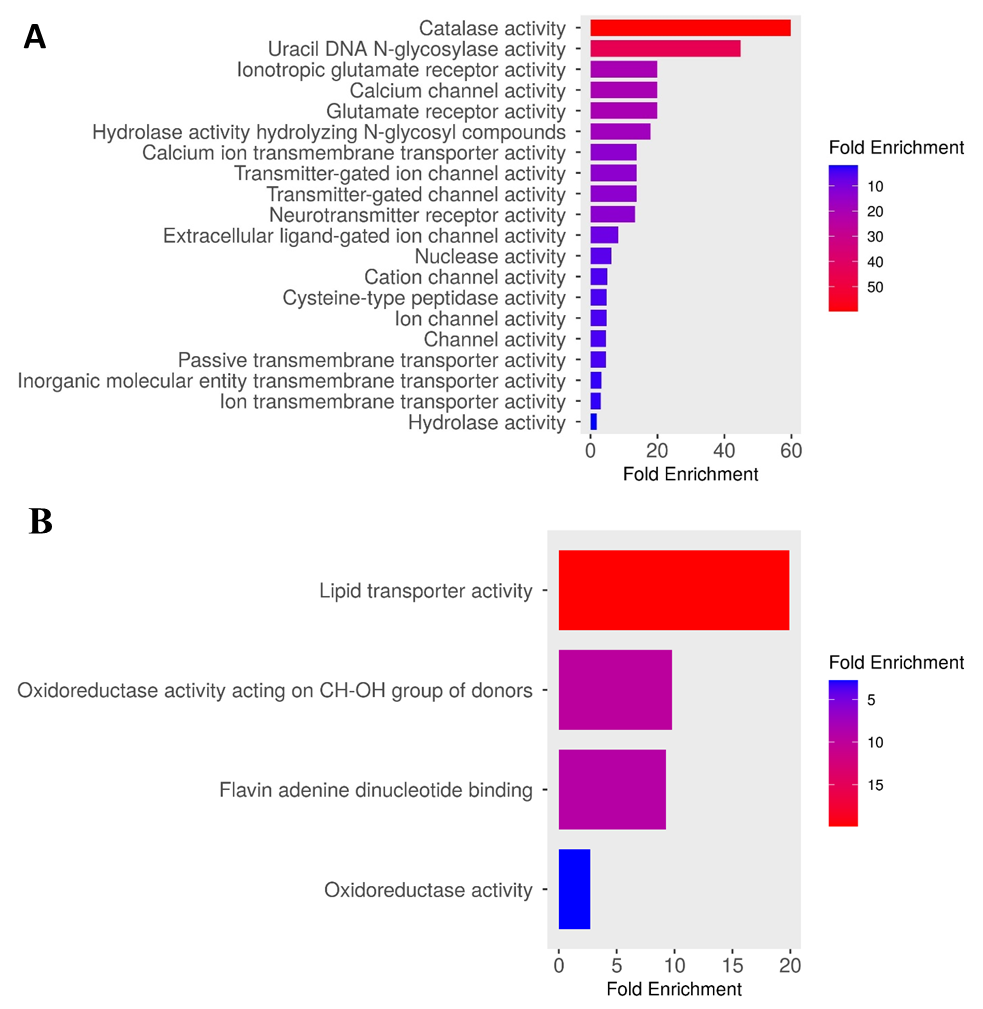


**S3 Fig. Molecular pathway enrichment analysis of differentially expressed transcripts between two field populations of CPB.** A) molecular pathways enriched in OFP; B) molecular pathways enriched in CFP.
